# Victims of Conspiracies? An Examination of the Relationship Between Conspiracy Beliefs and Dispositional Individual Victimhood

**DOI:** 10.1002/ejsp.70008

**Published:** 2025-07-17

**Authors:** Daniel Toribio-Flórez, Marlene S. Altenmüller, Karen M. Douglas, Mario Gollwitzer, Indro Adinugroho, Mark Alfano, Denisa Apriliawati, Flavio Azevedo, Cornelia Betsch, Olga Białobrzeska, Amélie Bret, André Calero Valdez, Viktoria Cologna, Gabriela Czarnek, Sylvain Delouvée, Kimberly C. Doell, Simone Dohle, Dmitrii Dubrov, Małgorzata Dzimińska, Christian T. Elbaek, Matthew Facciani, Antoinette Fage-Butler, Marinus Ferreira, Malte Friese, Simon Fuglsang, Albina Gallyamova, Patricia Garrido-Vásquez, Mauricio E. Garrido Vásquez, Oliver Genschow, Omid Ghasemi, Theofilos Gkinopoulos, Claudia González Brambila, Hazel Clare Gordon, Dmitry Grigoryev, Alma Cristal Hernández-Mondragón, Tao Jin, Sebastian Jungkunz, Dominika Jurgiel, John R. Kerr, Lilian Kojan, Elizaveta Komyaginskaya, Claus Lamm, Jean-Baptiste Légal, Neil Levy, Mathew D. Marques, Sabrina J. Mayer, Niels G. Mede, Taciano L. Milfont, Panagiotis Mitkidis, Jonas P. Nitschke, Mariola Paruzel-Czachura, Michal Parzuchowski, Ekaterina Pronizius, Katarzyna Pypno-Blajda, Gabriel Gaudencio Rêgo, Robert M. Ross, Philipp Schmid, Samantha K. Stanley, Stylianos Syropoulos, Ewa Szumowska, Claudia Teran-Escobar, Boryana Todorova, Iris Vilares, Izabela Warwas, Marcel Weber, Mareike Westfal, Adrian Dominik Wojcik

**Affiliations:** 1 School of Psychology, University of Kent, Canterbury, UK; 2 Department of Psychology, Ludwig-Maximilians-Universität München, Munich, Germany; 3 Leibniz Institute for Psychology, Trier, Germany; 4 Department of Psychology, University of Sheffield, Sheffield, UK; 5 Faculty of Psychology, Atma Jaya Catholic University of Indonesia, Jakarta, Indonesia; 6 Department of Philosophy, Macquarie University, Sydney, Australia; 7 Department of Psychology, Universitas Islam Negeri Sunan Kalijaga, Yogyakarta, Indonesia; 8 Department of Interdisciplinary Social Studies, University of Utrecht, Utrecht, the Netherlands; 9 Institute for Planetary Health Behaviour, University of Erfurt, Erfurt, Germany; 10 Department of Psychology, SWPS University of Social Sciences and Humanities, Warsaw, Poland; 11 Department of Psychology, Nantes Université, LPPL, Nantes, France; 12 Institute for Multimedia and Interactive Systems, University of Lübeck, Lübeck, Germany; 13 Department of the History of Science, Harvard University, Cambridge, Massachusetts, USA; 14 Institute of Psychology, Jagiellonian University, Kraków, Poland; 15 Laboratoire de Psychologie (LP3C), Université Rennes 2, Rennes, France; 16 Department of Cognition, Emotion, and Methods in Psychology, Faculty of Psychology, University of Vienna, Vienna, Austria; 17 Institute of General Practice and Family Medicine, University of Bonn, University Hospital Bonn, Bonn, Germany; 18 Center for Sociocultural Research, HSE University, Moscow, Russia; 19 Department of Labour and Social Policy, University of Lodz, Lodz, Poland; 20 Department of Management, Aarhus University, Aarhus, Denmark; 21 Department of Computer Science and Engineering, University of Notre Dame, Notre Dame, Indiana, USA; 22 School of Communication and Culture, Aarhus University, Aarhus, Denmark; 23 Department of Psychology, Saarland University, Saarbrücken, Germany; 24 Department of Political Science, Aarhus University, Aarhus, Denmark; 25 Department of Psychology, Universidad de Concepción, Concepción, Chile; 26 Institute for Management & Organization, Leuphana University, Lueneburg, Germany; 27 UNSW Institute for Climate Risk & Response, University of New South Wales, Sydney, Australia; 28 Behavior in Crisis Lab, Institute of Psychology, Jagiellonian University, Krakow, Poland; 29 Department of Business, Instituto Tecnológico Autónomo de México, Mexico City, Mexico; 30 Department of Research and Multidisciplinay Studies, Centro de Investigación y de Estudios Avanzados del Instituto Politécnico Nacional, Instituto Politécnico Nacional de México, Mexico City, Mexico; 31 Department of Psychology, University of Minnesota, Minneapolis, Minnesota, USA; 32 Chair of Political Sociology, University of Bamberg, Bamberg, Germany; 33 Institute of Political Science and Sociology, University of Bonn, Bonn, Germany; 34 Doctoral School of Social Sciences, Nicolaus Copernicus University, Toruń, Poland; 35 Department of Public Health, University of Otago, Wellington, New Zealand; 36 Laboratoire Parisien de Psychologie Sociale, Université Paris Nanterre, Nanterre, France; 37 Uehiro Centre for Practical Ethics, University of Oxford, Oxford, UK; 38 School of Psychology and Public Health, La Trobe University, Melbourne, Australia; 39 Department of Communication and Media Research, University of Zurich, Zurich, Switzerland; 40 School of Psychological and Social Sciences, University of Waikato, Tauranga, New Zealand; 41 Institute of Psychology, University of Silesia in Katowice, Katowice, Poland; 42 Penn Center of Neuroaesthetics, University of Pennsylvania, Philadelphia, Pennsylvania, USA; 43 Social and Cognitive Neuroscience Laboratory, Mackenzie Presbyterian University, São Paulo, Brazil; 44 Centre for Language Studies, Radboud University, Nijmegen, the Netherlands; 45 School of Psychology, University of New South Wales, Sydney, Australia; 46 School of Medicine and Psychology, The Australian National University, Canberra, Australia; 47 School of Sustainability, College of Global Futures, Arizona State University, Tempe, Arizona, USA; 48 Department of Psychology, Université Paris Nanterre, Nanterre, France; 49 Institute of Psychology, Nicolaus Copernicus University, Toruń, Poland

**Keywords:** conspiracy beliefs, conspiracy theories, victim justice sensitivity, victimhood

## Abstract

Conspiracy beliefs have been linked to perceptions of collective victimhood. We adopt an individual perspective on victimhood by investigating the relationship between conspiracy beliefs and the individual disposition to perceive and react to injustice as a victim, i.e., *victim justice sensitivity* (VJS). Data from two German samples (Ns = 370, 373) indicated a positive association between VJS and conspiracy mentality beyond conceptually related covariates (e.g., mistrust). In a multinational sample from 15 countries (*N* = 14,978), VJS was positively associated with both general and specific conspiracy beliefs (about vaccines and climate change) within countries, though these associations varied across countries. However, economic, sociopolitical and cultural country-level factors that might explain the cross-country variability (e.g., GDP, Human Freedom Index, individualism–collectivism), including indices of collective exposure to direct violence, did not moderate the studied associations. Future research should investigate the relationship between victimhood and conspiracy beliefs, considering both intraindividual and intergroup perspectives.

## Introduction

Conspiracy beliefs attribute specific events or circumstances of public interest to malevolent plots orchestrated by secret groups (Douglas and Sutton 2023). While conspiracy beliefs often highlight the malevolent intentions of powerful elites, they also underline the experience of victims suffering the secrecy and negative consequences of the presumed conspiracies (van der Linden 2023). For example, conspiracy beliefs related to vaccines assert that the public is victim of attempts at mass psychological manipulation or the deliberate spread of illnesses to control the population (e.g., ‘chemtrail vaccines’; Coleman 2022). Also, the narrative of current climate change conspiracy beliefs portrays citizens as victims of global elite plans to restrict individual freedoms in the name of climate action (e.g., ‘15-minute cities’ conspiracy theories; Paddison 2023). Thus, it is common for conspiracy believers to perceive and present themselves as victims of the secret plots they believe in (Lewandowsky et al. 2015).

In fact, conspiracy beliefs have been linked with a sense of *collective victimhood* (Bilewicz 2022). This refers to the psychological experience and consequences of a harm inflicted by one (perpetrating) group to another (victimised) group (Noor et al. 2017), usually operationalised as the individual perception of that harm inflicted on one’s ingroup. Research has demonstrated that individuals from groups who have suffered from historical trauma in the form of direct or structural violence (e.g., war, genocide, structural discrimination or repression) and who have consequently internalised a sense of victimhood in their group identity (Bilewicz 2022) endorse conspiracy beliefs more strongly (Bertin and Delouvée 2021; Bilewicz et al. 2019; Mashuri and Zaduqisti 2014; Nelson et al. 2010; Pantazi et al. 2022). In such contexts of historical trauma, conspiracy beliefs may serve as an adaptive mechanism, helping individuals from victimised groups to justify and cope with the conditions of collective victimhood, and potentially motivate a reaction against the outgroups perceived to be responsible for their collective trauma (e.g., a powerful elite; Bilewicz 2022; Bilewicz and Liu 2020).

Complementing this theorising and empirical evidence, we argue that the analysis of the role that victimhood plays in the endorsement of conspiracy theories should not be limited to collective victimhood but should also incorporate an *individual* dimension. Research suggests that conspiracy beliefs are indeed associated with perceptions of individual victimhood (Armaly and Enders 2022; Bertin 2024; Gkinopoulos and Mari 2023). Here, we introduce a dispositional perspective, focusing on whether people’s interindividual differences in *victim justice sensitivity* (VJS)—that is, an individual’s predisposition to perceive and emotionally react as a victim of injustice (Baumert and Schmitt 2016; Gollwitzer et al. 2013; Schmitt et al. 2005, 2010)—contributes to the understanding of conspiracy beliefs.

### 1.1 The Importance of Individual Victimhood for Conspiracy Beliefs

Beyond the introductory argument that conspiracy beliefs are narratives about victimhood, there are multiple reasons to consider individual victimhood as a relevant explanatory factor of conspiracy beliefs.

A first, perhaps obvious, but nevertheless important reason is that a person’s individual sense of victimhood is not exclusively related to group-level injustice. Individuals can be, or perceive themselves as, victims of unfairness in their daily lives (e.g., cheating, betrayal, bullying), irrespective of their belonging to a specific collective and its historical background (Mikula et al. 1990). For some people, such interpersonal experiences might constitute ‘critical life events’ that contribute to stabilising their individual predisposition to perceive and react against injustice from a victim’s perspective (Bondü et al. 2016; Gollwitzer et al. 2015). This individual predisposition could manifest in the endorsement of narratives like conspiracy theories that support the perception of oneself as a victim. Indeed, some of these experiences have been observed to be associated with the endorsement of conspiracy theories (e.g., bullying; Jolley and Lantian 2022).

Second, an individual sense of victimhood is conceptually aligned with idiosyncratic features of the self-protective psychology of conspiracy beliefs. Conspiracy beliefs have been associated with psychological motives related to the defence and enhancement of the individual self (Biddlestone et al. 2021), such as narcissistic tendencies (Cichocka et al. 2016), a need for uniqueness (Lantian et al. 2017) and a need for control (van Prooijen and Acker 2015). Importantly, individuals with a heightened self-consciousness and defensive self-evaluation, like conspiracy believers, are prone to interpersonal paranoia, as they tend to perceive themselves as the targets of other people’s behaviour (Cichocka et al. 2016). In some cases, this might reflect an acute distrust in interpersonal relationships and towards institutions (Mancosu et al. 2023; Meuer and Imhoff 2021; van Prooijen et al. 2022). This suspicious, self-protective mindset that characterises conspiracy believers has also been theorised to characterise the sensitivity toward individual victimhood, with those prone to perceive themselves as victims of others’ behaviour showing a tendency to distrust and cooperate less with others (Gollwitzer et al. 2013).

A third important reason is that variables related to the individual self (e.g., individual narcissism, need for uniqueness) are stronger predictors of conspiracy beliefs relative to those associated with the collective self (e.g., collective narcissism, collective victimhood), according to recent systematic and meta-analytic reviews (Biddlestone et al. 2021; Biddlestone, Green, et al. 2022). One plausible explanation for this is that factors associated with the collective self might only be relevant when people’s group identity is salient, and specific conspiracy beliefs are adopted to enhance their ingroup image by negatively portraying the outgroup as evil conspirators (Biddlestone, Green, et al. 2022). This also seems to be the case for victimhood: when sharing a sense of collective victimhood, only individuals with a strong group identity are more inclined to embrace conspiracy beliefs (Pantazi et al. 2022).

### 1.2 From Perceived to Dispositional Individual Victimhood

Thus far, evidence of the association between individual victimhood and conspiracy belief is limited. There are several indicators indirectly suggesting that conspiracy beliefs are related to individual victimhood. For example, conspiracy beliefs are associated with proxies of individual victimhood, such as individual socioeconomic precarity (Adam-Troian et al. 2023; Adamus et al. 2024) and perceived economic inequality (Salvador Casara et al. 2022). More directly, recent cross-sectional and experimental evidence indicates that conspiracy beliefs are positively associated with individual perceptions of victimhood in specific national contexts (Armaly and Enders 2022) and that conspiracy beliefs can increase these perceptions (Bertin 2024). While this evidence highlights that conspiracy beliefs influence perceived victimhood, methodological limitations make it premature to rule out a bidirectional reinforcing association between victimhood and conspiracy beliefs (Bertin 2024). In fact, secondary analyses of the effect of primed individual victimhood on conspiracy beliefs suggest that such bidirectional causality could be warranted (Gkinopoulos and Mari 2023).

Critically, an important aspect that the available research has not considered is that people differ in their individual predisposition to perceive and react against injustice as victims. Moving beyond the conceptualisation of individual victimhood as a temporal perception or ‘psychological state’ (Armaly and Enders 2022), we investigated whether people’s more stable, dispositional sensitivity towards individual victimhood (i.e., VJS) is related to conspiracy beliefs.

VJS is conceptualised as a trait-like dispositional variable capturing individual differences in the tendency to perceive and emotionally react against being exploited by others and becoming a victim of injustice (Schmitt et al. 2010). Individuals high in VJS are hypervigilant towards cues of untrustworthiness, and they tend to be suspicious and distrusting of others—even members of their ingroup (Altenmüller et al. 2023; Gollwitzer et al. 2021)— to avoid being exploited (Gollwitzer and Rothmund 2009, 2011). VJS is especially influential in uncertain, ambiguous and complex situations, where cues of untrustworthiness are more salient for individuals with high VJS (Baumert et al. 2011; Gollwitzer and Rothmund 2009, 2011). Moreover, VJS has been linked to a low sense of, but high need for, control (Baumert et al. 2014; Baumert and Schmitt 2016; Buchholz et al. 2023). Accordingly, it is likely that VJS could play an important role in the endorsement of conspiracy theories, particularly given their associations with generalised distrust (Thielmann and Hilbig 2023; van Prooijen et al. 2022) and their function in reducing the complexity of, and restoring feelings of control over, uncertain or ambiguous social contexts (van Prooijen and Acker 2015; van Prooijen and Douglas 2017; van Prooijen and Jostmann 2013). Beyond their psychological correlates, VJS and conspiracy beliefs are likely connected through people’s political orientation. The self-oriented tendency characterizing VJS has been argued to contribute to the foundation of (right-wing) populist attitudes (Rothmund et al. 2017, 2020), while populism is one of the strongest political correlates of conspiracy beliefs in specific national contexts (e.g., Uscinski et al. 2022).

## 2 Research Overview

In the present research, we investigated whether VJS, as the individual disposition to perceive and emotionally react as a victim of injustice, is positively related to conspiracy beliefs. We conducted two studies. In Study 1, we performed secondary analyses of data from Altenmüller and Poppe (2025) to examine the association between VJS and conspiracy mentality—conceptualised as the general tendency to endorse conspiracy theories (Bruder et al. 2013; Milošević Đorđević, Žeželj, et al. 2021)—while controlling for conceptually relevant covariates. In Study 2, we collected large-scale, cross-national survey data within a ManyLabs research project to examine the association between VJS and both general and specific conspiracy beliefs, as well as to explore the generalisability and heterogeneity of these associations across 15 different countries.

## 3 Study 1—Secondary Analyses of Altenmüller and Poppe (2025)

To examine the association between individual victimhood and conspiracy beliefs beyond their common correlates (e.g., general mistrust), we conducted secondary analyses with data from German samples made openly available by Altenmüller and Poppe (2025). In two studies, the researchers investigated the role of different trait-like variables for motivated science perception, including VJS and conspiracy mentality. Participants were presented with articles describing specific scientific findings which were more or less (in)congruent with their own pre-existing attitudes towards the topic in question and then reported their trust in the reported science. Of particular interest for the present research, participants also responded to scales assessing VJS and conspiracy mentality, and, in the second study, to a range of other related dispositional measures (i.e., dispositional mistrust, intolerance of ambiguity and need for control). Here, we report our reanalysis of these data, exploring the relationship between VJS and conspiracy mentality.

### 3.1 Method

#### 3.1.1 Participants

Sample 1 consisted of 370 German participants (from the general and student population; 69% female; 17–63 years old, *M* = 33.61, SD = 15.47), while Sample 2 comprised 373 German participants (from the student population; 81% female; 17–63 years old, *M* = 22.75, SD = 4.95). Sensitivity analyses indicated that these sample sizes enabled the detection of correlations as small as *r* = 0.17 with 90% power, assuming *α* = 0.05.

#### 3.1.2 Measures

VJS was measured through the VJS 10-item scale from the *Justice Sensitivity Inventory* (Schmitt et al. 2010; e.g., ‘I cannot easily bear it when others profit unilaterally from me’), whereas conspiracy mentality was measured using the *Conspiracy Mentality Questionnaire* (Bruder et al. 2013; e.g., ‘I think many very important things happen in the world, which the public is never informed about’). Of note, while Altenmüller and Poppe (2025) used an extended measure of conspiracy mentality to better fit the context of their specific stimuli, here we focus on the validated conspiracy mentality scale consisting of five items. For other measures and covariates, see Altenmüller and Poppe (2025). All scales ranged from 1 to 6.

### 3.2 Results

Analyses with each sample showed a small-to-moderate positive correlation between VJS and conspiracy mentality. In Sample 1 (*n* = 370), the correlation was *r* = 0.12, 95% CI [0.02, 0.22], *p* = 0.021, and in Sample 2 (*n* = 373), it was *r* = 0.20, 95% CI [0.10, 0.30], *p* < 0.001. Importantly, with the data from Sample 2, we further regressed VJS on conspiracy mentality while accounting for the association with covariates that we argued are conceptually linked to both VJS and conspiracy beliefs, that is, dispositional mistrust, intolerance of ambiguity, need for control and political orientation. The association between VJS and conspiracy mentality remained statistically significant (see Table 1), which suggests that the small-to-moderate positive association held above and beyond some of their conceptually relevant correlates.

Taken together, Study 1 showed with two German samples that VJS was weakly to moderately associated with conspiracy mentality, beyond their shared association with people’s dispositional distrust, their (in-)tolerance to ambiguity, their need for control and their political orientation. Despite the supportive results, Study 1 was limited in terms of cross-cultural generalisability and the assessment of specific conspiracy beliefs, issues that were addressed in the next study.

## 4 Study 2—TISP Cross-National Survey

In Study 2, we further investigated the association between VJS and general and specific conspiracy beliefs through a large-scale, cross-national dataset from the *Trust in Science and Science-Related Populism* (TISP) ManyLabs research project (a multinational, crowd-sourced research collaboration to examine the correlates of trust in scientists and science-related populist attitudes across countries; Cologna et al. 2025). Examining the association between VJS and conspiracy beliefs via a cross-national sample had two main advantages. First, it enabled a more robust test of the generalisability of the hypothesised association by analysing average within-country trends. Second, it allowed us to assess cross-country differences and explore the potential moderating role of economic, sociopolitical and cultural factors that could modulate the strength of the association between VJS and conspiracy beliefs; some of these country-level factors have been previously observed to be associated with conspiracy beliefs (Hornsey, Pearson, et al. 2023; Alper and Imhoff 2022; Cordonier et al. 2021). For example, countries’ economic wealth and (in-)equality—e.g., gross domestic product (GDP), Gini index of economic inequality (Hornsey et al. 2023; Salvador Casara et al. 2022)—, the guarantee and protection of personal, political or economic freedoms, as well as trust in the institutional system that embodies these freedoms—i.e., indices of democratic health and institutional trust (Cordonier et al. 2021; Hornsey and Pearson 2022; van Prooijen et al. 2022)—, could impact the sense of individual powerlessness and disenfranchisement that justifies the experience of individual victimhood and underlies the endorsement of conspiracy beliefs. Similarly, cultural differences in the degree of individualistic–collectivistic values should warrant a more or less self-oriented sensitivity towards injustice, which could reflect in a higher or lower endorsement of conspiracy narratives (Hornsey and Pearson 2022). Last but not least, country-level indicators of involvement in intergroup conflicts and of state political terror could help clarify whether the expected association between VJS and conspiracy beliefs may differ in countries with a history of direct violence, this being external or internal; after all, in these countries a sense of collective victimhood may be an internalised feature of the collective identity (Bilewicz 2022), which may cultivate a sense of individual-level victimhood and fuel conspiratorial thinking accordingly (e.g., Bertin and Delouvée 2021; Bilewicz et al. 2019; Pantazi et al. 2022).

Moreover, in this study, we used measures of both general and specific conspiracy beliefs, which may inform current debates concerning the distinction between these two constructs—particularly in terms of their conceptual definition and different correlates—and their presumed causal relationship (Imhoff et al. 2024; Sutton et al. 2024). In this study, specific beliefs referred to *climate change* and *vaccine* conspiracy beliefs, which have received much attention in recent research (e.g., Biddlestone, Azevedo, et al. 2022; Chen et al. 2021; Milošević Đorđević, Mari, et al. 2021) and aligned with the overall research goals and content of the TISP project.

### 4.1 Method

#### 4.1.1 Ethics and Open Science

The study was conducted following APA standards. The TISP ManyLabs research project was considered exempt from full IRB review from the Harvard University—Area Committee on the Use of Human Subjects (protocol #IRB22-1046) in August 2022. A modified IRB application was submitted and considered exempt from full IRB review by the Harvard University—Area Committee on the Use of Human Subjects in November 2022 (protocol #IRB22-1046). All authors obtained IRB approval when required by their institutions, especially considering the inclusion of the measures of conspiracy beliefs and VJS, which were not part of the original TISP IRB application.

The sampling procedure, research materials, hypothesis and analysis plan were preregistered prior to data analyses (https://osf.io/btsn5/). Although data collection had started at the time of the preregistration, only one out of the 15 countries comprising our final sample had completed the data collection, and these data had not been analysed before finalising the preregistration.

In the present research, we exclusively focus on Hypothesis 3 of the preregistration—’Victim sensitivity is positively associated with (H3.1) general conspiracy beliefs (i.e., CT_lantian) and specific (H3.2) climate-related (i.e., CT_climate) as well as (H3.3) vaccine-related conspiracy beliefs (i.e., CT_vaccine)’. The reason for this is that the other hypotheses refer to a different research question more closely related to the main TISP research project, specifically regarding the association between VJS and trust in science (H1) and science-related populism (H2), that will be reported independently. Data and code for reproducing the reported analyses, as well as translated measures, are available at the Open Science Framework (https://osf.io/72dcn/). For details on the preparation of the dataset, see Supporting Information, as well as the description of the TISP dataset in Mede et al. (2025).

#### 4.1.2 Participants

The online multinational TISP survey was programmed in Qualtrics, translated into each country’s official language, with participant recruitment managed by panel providers (in most countries, Bilendi & Respondi; https://www.bilendi.co.uk/) between December 2022 and March 2023. The data were collected by means of balanced gender × age quotas and were weighted based on national distributions of age, gender and education level, as well as country sample size using post-stratification (for more details, see Supporting Information and Mede et al. 2025).

The present study is based on a subsample from the TISP project, which, in addition to the TISP main survey, answered a set of secondary measures assessing conspiracy beliefs and VJS (i.e., 47,419 participants from *k* = 16 countries, before exclusions). This subsample was determined by the availability of each group of collaborators to include the list of secondary measures in their national survey. From this subsample, we first excluded participants who did not complete the survey, either because they did not accept the informed consent (i.e., 1770), because they withdrew from completing the survey (i.e., 4834) or because they were prevented from starting the survey as they exceeded the gender × age quotas (i.e., 13,757). Second, we excluded participants based on two preregistered attention checks included in the main survey. Note that we preregistered that we would exclude participants who failed the two attention checks after data collection, but this was not possible because the original TISP survey had been programmed to automatically prevent participants from continuing after failing one of the attention checks. Thus, effectively, participants were excluded right after failing any of the two attention checks (i.e., 1806 failed the first check, ‘Please write the number 213 into the comment box,’ and 7233 failed the second check, ‘To show us that you are still paying attention, please select ‘strongly disagree”; for failing rates per country, see Table S1). In those countries where panel providers successfully shared participants’ panel IDs, we further excluded duplicate responses from participants with the same panel ID (i.e., 28), keeping only the first complete response for each duplicate. In preparation to calculate the post-stratification weights, we further had to exclude all participants with missing values in any of the post-stratification variables (i.e., 66 cases with missing gender, age and education). Since the dataset we used to obtain national gender distributions for the calculation of post-stratification weights did not include data regarding non-binary gender identities (i.e., World Population Prospects 2022 of the United Nations; UN 2022), we further excluded participants who did not identify as female or male (i.e., 329). Additionally, we excluded part of the data from three countries (i.e., Brazil, *n* = 1117; Germany, *n* = 1000; New Zealand, *n* = 501), due to discrepancies in the Likert scales used for key measures, which differed from those specified below and applied by most national subsamples. However, for Germany and New Zealand, we retained data collected using the correct Likert scales from other groups of collaborators. Thus, the final sample for the study consisted of 14,978 participants from *k* = 15 countries (for demographics, see Table 2).

Based on the collected data, we conducted simulations to estimate the statistical power to detect different sizes of the association between VJS and the different measures of conspiracy beliefs in our preregistered multilevel models (see below). Our final sample size was sufficiently large to detect significant unstandardised regression coefficients as small as *b* = 0.15, in the case of our measures of *climate* and *vaccine* conspiracy beliefs, and as small as *b* = 0.20, in the case of our measure of *general* conspiracy beliefs, with sufficient statistical power (1 − *β* ≥ 0.845). For further details, see Supporting Information.

#### 4.1.3 Procedure and Measures

Participants provided informed consent and completed the TISP main questionnaire, including demographic variables such as age, gender, education, residence setting (urban vs. rural), political orientation and religiosity, as well as other variables unrelated to the purpose of the present study (for the complete list, see Mede et al. 2025). Once the TISP main questionnaire was completed, participants reported their agreement with two *specific* (science-related) conspiracy beliefs, one related to climate change (‘Climate change is a hoax orchestrated by climate scientists’) and one related to vaccines (‘Scientists work together to cover up the dangers of vaccines’; 1 = *strongly disagree*, 5 = *strongly agree*). They also answered the single-item measure of *general* conspiracy beliefs from Lantian et al. (2016; ‘I think that the official version of the events given by the authorities very often hides the truth’; 1 = *completely false*,9 = *completely true*). As in previous cross-national studies (Hornsey et al. 2023), the examples of conspiracy beliefs often included in the preamble of this scale were removed, as familiarity with some of them likely varies across countries (e.g., the death of Princess Diana) and could introduce measurement error. Finally, participants completed the VJS subscale from the short version of the *Justice Sensitivity Inventory* (Baumert et al. 2014), which consisted of two items (‘It makes me angry when others are undeservingly better off than me’ and ‘It worries me when I have to work hard for things that come easily to others’; 1 = *strongly disagree*, 6 = *strongly agree*; Spearman–Brown’s estimate *ρ* = 0.80, range across countries, *ρ*s = 0.72 − 0.86).

### 4.2 Results

#### 4.2.1 Confirmatory Analyses

To test the hypothesis that VJS would be positively associated with the different measures of conspiracy beliefs, we used a multilevel framework. Prior to our analyses, the measure of VJS was *centred within clusters* (CWC) by subtracting the country mean from participants’ VJS score; this way, VJS captured the variance around each country’s mean, not around the grand mean. This *centring within cluster* approach allowed us to examine the within-country association between VJS and conspiracy beliefs (Enders and Tofighi 2007). As preregistered, we fitted three different multilevel models with random slopes. Each model respectively included one of the measures of conspiracy beliefs (i.e., CB climate, CB vaccine, CB general) as dependent variable and VJS (CWC) as fixed effect. We observed that, on average, there were significant positive within-country associations between VJS and the different conspiracy beliefs across countries, although the strength of these associations was small across measures (see Table 3). Specifically, as indicated by the marginal *R*^*2*^, the fixed effect of VJS (CWC) contributed to explain 1.8% of the variance of climate change conspiracy beliefs, 3.9% of the variance of vaccine conspiracy beliefs and 5.3% of the variance of general conspiracy beliefs.

#### 4.2.2 Exploratory Analyses

##### 4.2.2.1 Withinvs. Between-Country Association

Our study did not include a measure of collective victimhood to isolate the association between VJS and conspiracy beliefs. However, the multilevel nature of the data enabled us to discriminate the extent to which conspiracy beliefs were associated with VJS at the individual level (i.e., *within-country* effect) and at the country, collective level (i.e., *between-country* effect). To explore this, we added the country-level mean of VJS as an additional fixed effect to the models described above (see Table 4). Furthermore, we used custom contrasts to inspect the difference between the between- and the within-country effects, which represents the *contextual* effect (Enders and Tofighi 2007). The contextual effect captures the part of the variance explained by the between-country effect that cannot be attributed to the within-country effect; in other words, how the average VJS of people from the same country is associated with individual conspiracy beliefs, over and above the contribution of individual VJS. For example, if two people from different countries have the same VJS, the contextual effect indicates how the difference in the average VJS of their respective countries (arguably, a proxy of their country’s collective sensitivity to victimhood) is associated with their individual conspiracy beliefs.

In the case of the measures CB climate and CB general, the between-country effects of VJS were statistically non-significant, as were the contextual effects (*b* = 0.19, SE = 0.18, *z* = 1.08, *p* = 0.280, *p*_Bonferroni–Holm_ = 0.561 and *b* = −0.14, SE = 0.49, *z* = −0.28, *p* = 0.780, *p*_Bonferroni–Holm_ = 0.780, respectively). Regarding CB vaccine, we observed a statistically significant between-country effect and a non-significant contextual effect (*b* = 0.54, SE = 0.25, *z* = 2.19, *p* = 0.029, *p*_Bonferroni–Holm_ = 0.086), suggesting that vaccine conspiracy beliefs were associated with the average country-level VJS but not over and above the association with individual-level VJS. These results suggest that the observed association between VJS and conspiracy beliefs mainly corresponds to the individual-level related to victimhood, rather than country-, collective-level processes.

Of note, when we further accounted for demographic variables including age, gender, residence setting, political orientation and religiosity, our results were generally similar (see Table 5). The only exception was the within-country association of VJS with CB climate, which did not reach statistical significance, possibly due to the lower statistical power and the association of CB climate with some of the demographic covariates (e.g., political orientation). When controlling for these variables in the preregistered analyses (without country-level VJS as a fixed effect), we found the same pattern of results (see Table S2).

##### 4.2.2.2 Examination of Cross-Country Heterogeneity

**Figure 1:**
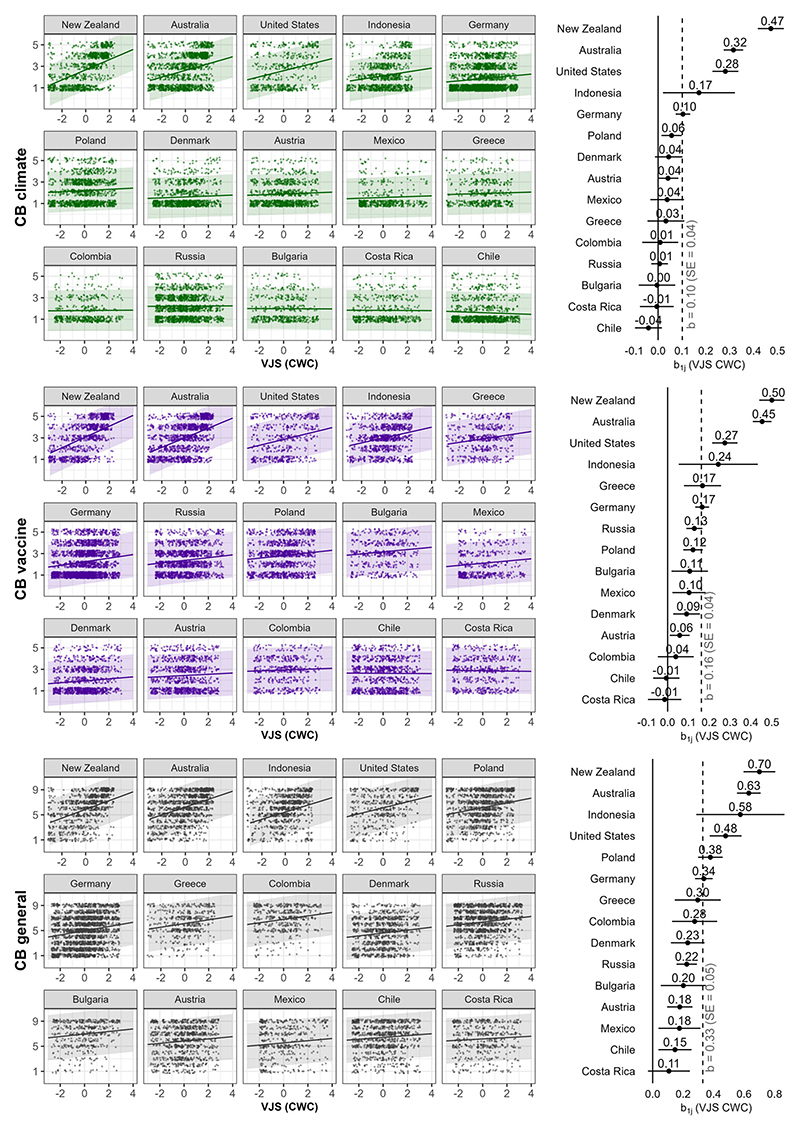
Random slopes (left) and regression weights (right) of victim justice sensitivity (VJS) on different measures of conspiracy beliefs across countries. *Note:* Bandwidth and error bars represent 95% CIs. The dashed vertical line denotes the average within-country association.

Moreover, we observed heterogeneity in the associations between conspiracy beliefs and VJS across countries (see Figure 1). More specifically, we observed that the associations between VJS and climate change and vaccine conspiracy beliefs varied from countries like Costa Rica, Chile and Colombia, where this association was virtually null, to countries like New Zealand, Australia and the United States, where it was moderately positive. Regarding general conspiracy beliefs, the association with VJS was positive in every country, yet it differed in strength from a weak association in countries like Costa Rica, Chile and Mexico to a moderate association in countries like New Zealand, Australia and Indonesia.

To test whether this level of between-country heterogeneity was statistically meaningful, we compared the fit of the preregistered random slopes models with a version of these models without country-level random slopes and only random intercepts. Across the three different measures of conspiracy beliefs, the models with country-level random slopes showed a significantly better model fit than the random-intercept models, confirming that the heterogeneity across countries was significantly meaningful (see Table S3).

We further explored the source of heterogeneity by examining the cross-level interactions between VJS and country-level variables reflecting economic, sociopolitical and cultural differences that could moderate the association between VJS and conspiracy beliefs. Among the economic and sociopolitical factors, we considered country-level variables that, besides being conceptually and empirically related to conspiracy beliefs (Hornsey et al. 2023; Alper and Imhoff 2022; Cordonier et al. 2021), could shift people’s perceptions of powerlessness, injustice and systemic failure, and therefore, moderate the VJS-conspiracy belief association. These country-level variables we examined were the countries’ *GDP per capita* (World Bank/OECD 2022a), the *Gini Index* of economic inequality (World Bank/OECD 2022b), the *Human Freedom Index* (Vásquez et al. 2022), the *Corruption Perception Index*^1^ (Transparency International 2022) and an index of *institutional trust* (World Values Survey; Haerpfer et al. 2022). Furthermore, we considered that cultural differences in *individualism/collectivism* (Hofstede et al. 2010) could also modulate the extent to which VJS is associated with conspiracy beliefs. Finally, we sought to explore whether the association between VJS and conspiracy beliefs was dependent on country-level indicators of direct violence, on the premise that the exposure to such violence may foster the internalisation of collective victimhood within the countries’ collective identity (Bilewicz 2022), and thereby, moderate the relationship between individual-level victimhood and conspiracy beliefs. Specifically, we used the number of *armed conflicts* a country had been involved in since 1946 (UPCD/PRIO 1946–2023 dataset; Davies et al. 2024; Gleditsch et al. 2002) as a measure of *external* direct violence, and the country-level scores in the *Political Terror Scale* (Gibney et al. 2024), a measure of state violation of physical and personal integrity rights to capture exposure to *internal* violence. For a more detailed description of all country-level indexes, see the Supporting Information.

As noted by Hornsey et al. (2023), country-level variables tend to be highly correlated with each other. This was also the case in our data (see Figure S2), which could entail problems of multicollinearity. Thus, we explored the cross-level interaction between VJS and each country-level predictor independently, fitting a total of 27 independent random slopes models. Each model included the respective measure of conspiracy beliefs as a dependent variable (i.e., CB climate change, CB vaccine or CB general), and VJS (CWC), the respective country-level predictor centred at the grand mean and their cross-level interaction term as fixed effects (see Tables S4–S12). The models further accounted for the VJS (CWC) random slope variability across countries.

Across every model, we observed that the average within-country association between VJS and each respective measure of conspiracy beliefs was statistically significant. We did not observe any statistically significant effect of the country-level predictors or cross-level interactions. The only exception was a significant cross-level interaction between VJS and Hofstede’s individualism–collectivism index (Hofstede et al. 2010), showing that the VJS-conspiracy beliefs association was stronger in more individualistic countries. However, this cross-level interaction did not replicate when using two other, more recent and less biased indices of individualism–collectivism (Minkov and Kaasa 2022; Pelham et al. 2022). Taken together, our exploratory results suggest that there is between-country heterogeneity in the relationship between VJS and conspiracy beliefs. However, this heterogeneity can neither be generally attributed to conceptually relevant economic, sociopolitical and cultural countrylevel factors nor to indices of collective exposure to direct violence.

## 5 Discussion

Research has addressed the link between conspiracy beliefs and victimhood with a particular focus on how *collective* victimhood can be an antecedent of conspiracy beliefs (e.g., Bilewicz 2022; Pantazi et al. 2022). Recent findings indicate that temporal (state-like) perceptions of *individual* victimhood are also a correlate and potential consequence of conspiracy beliefs (Armaly and Enders 2022; Bertin 2024). Here, we focus on whether people’s trait-like individual predisposition for individual victimhood is associated with their conspiracy beliefs. The current research offers supportive evidence of a small-to-moderate, positive association between conspiracy beliefs and VJS (Gollwitzer et al. 2013; Schmitt et al. 2005, 2010), that is, the individual difference in people’s disposition to perceive themselves (rather than the group) as victims of injustice and to react emotionally and behaviourally to this (presumed) self-oriented injustice. In Study 1, we found that German respondents with higher VJS showed stronger conspiracy mentality, over and above common correlates such as dispositional mistrust, intolerance of ambiguity and need for control. In Study 2, through the analysis of large-scale crossnational data, we further observed that individual VJS is associated with higher endorsement of different conspiracy beliefs (i.e., that climate change is a hoax orchestrated by scientists, that scientists are hiding the dangers of vaccines and that the official account of certain events shared by authorities very often hides the truth). These associations remained significant for vaccinerelated and general conspiracy beliefs when controlling for broad variables such as demographics (age, gender, residence setting), political orientation and religiosity. These findings suggest that the conceptual association between victimhood and conspiracy beliefs should further consider the role of people’s individual predisposition to perceive themselves and react as victims.

Importantly, we observed that the association between conspiracy beliefs and the countries’ average VJS was not significant beyond their association with individual VJS, suggesting that conspiracy beliefs are primarily associated with people’s individual predisposition to perceive and react as victims of injustice, and not with the average predisposition of their fellow nationals. Furthermore, we did not find country-level indices of collective exposure to direct violence (i.e., involvement in armed conflicts, state political terror) to moderate the association between VJS and conspiracy beliefs, suggesting that this association held irrespective of contextual features potentially linked to a sense of collective victimhood (Bilewicz 2022; Noor et al. 2017). Even a descriptive inspection of the effect sizes across those countries in our sample that are considered to have experienced processes of collective victimhood (e.g., Poland, Greece, Mexico, Chile) shows that the association of VJS and conspiracy beliefs is not necessarily smaller or lager compared to other countries (e.g., Germany, Austria). Taken together, the present findings indicate that the association between conspiracy beliefs and dispositional individual victimhood is warranted beyond possible associations with collective victimhood.

The link between VJS and conspiracy beliefs can first be conceptualised from an individual psychological perspective. Individuals high in VJS may be particularly drawn to conspiracy theories because such narratives reinforce a worldview in which one is the target of secret, nefarious plots. This alignment may serve a cognitive consistency function, allowing individuals to maintain a coherent self-image rooted in their stable disposition to perceive themselves as victims of injustice. Individuals high in VJS also tend to process ambiguous information in justice/injustice terms (Baumert et al. 2012) and infer hostility in other people’s ambiguous behaviour (Bondü 2018). Thus, it is plausible that, under ambiguity, VJS introduces attributional bias toward external, potentially conspiratorial, causes of injustice. Furthermore, the self-protecting features characterising people with high VJS under the possibility of being treated unfairly (i.e., self-oriented tendencies and interpersonal distrust; Baumert and Schmitt 2016) overlap conceptually with well-documented correlates of conspiracy beliefs, like narcissism, need for uniqueness and interpersonal and institutional distrust (Douglas and Sutton 2023). Importantly, we argued that people’s VJS may stem from critical personal experiences of injustice (Bondü et al. 2016; Gollwitzer et al. 2015), not necessarily associated with group memberships. Thus, individuals high in VJS may still be inclined to endorse conspiracy theories as a psychological means of explaining, justifying or externalising their sense of grievance, irrespective of the presence or absence of a sense of collective victimhood (e.g., in countries like the United States).

This said, the broader sociocultural context may still shape how the observed association between VJS and conspiracy beliefs unravels. Indeed, the exploratory results of Study 2 revealed cross-country differences in the studied association, but our findings are unclear regarding what this heterogeneity can be attributed to. We examined whether relevant economic, sociopolitical, cultural and historical country-level factors (i.e., GDP per capita, Human Freedom Index, institutional trust, individualism–collectivism, exposure to direct violence) moderated the association between VJS and conspiracy beliefs. For example, we considered that a country’s economic wealth and protection of freedom should affect people’s perceptions of powerlessness, which might justify the experience of individual victimhood and the belief in conspiracy theories. Similarly, cultural differences in individualism–collectivism should warrant a self-oriented sensitivity toward injustice that is reflected in the endorsement of conspiracy theories. We further considered that in societies with a history of direct violence (e.g., involvement in armed conflicts or state political terror), individuals may share a sense of collective victimhood that influences the relationship between VJS and conspiracy beliefs. However, we did not consistently find any significant cross-level interactions between the considered country-level predictors and people’s VJS. These null findings point to potential limitations in our selection of country-level indicators (see below for a discussion on the limitations of country-level indices of direct violence). Future research should explore alternative country-level predictors that the present work may have overlooked—such as societal polarisation or narratives of collective trauma—that might directly reflect the emotional and potentially symbolic foundations of people’s sense of victimhood to better account for the cross-country heterogeneity in the VJS-conspiracy beliefs association.

The present research responds to recommendations to integrate different levels of analysis in the study of conspiracy beliefs (Hornsey et al. 2023). The findings of our cross-national study provide preliminary evidence that, at the *micro* or individual level, people’s individual sense of victimhood is associated with conspiracy beliefs, above and beyond country-level effects of individual victimhood. While at the *meso* or intergroup level, a strong identification with a collective that suffered negative historical experiences has been shown to be related to and increase conspiracy beliefs (Bilewicz et al. 2019; Mashuri and Zaduqisti 2014; Pantazi et al. 2022), at the micro or individual level, people’s individual disposition to feel and react as victims of injustice might also play an important role regarding the endorsement of conspiracy theories. These two factors are not independent but are likely not totally codependent either. For example, identification with the ingroup might play a role in how VJS impacts individual-level behaviours (Baumert et al. 2020; Magraw-Mickelson et al. 2022; Süssenbach and Gollwitzer 2015). Future research should clarify the extent to which, and under which conditions, individual victimhood explains the endorsement of conspiracy theories, and how this relationship might interact with people’s sense of collective victimhood. Additionally, to draw any conclusions about the directionality of this relationship, it is important to conduct further experimental or longitudinal research. So far, the only published experimental research suggests that exposure to conspiracy beliefs can increase perceived individual victimhood; however, it does not rule out a bidirectional causal association (Bertin 2024). While conspiracy beliefs can indeed play a victimising role by increasing perceptions of individual victimhood, the individual trait-like tendency to feel and react like a victim can still be associated with conspiracy beliefs.

The different results and effect sizes observed across distinct conspiracy beliefs we measured suggest that the association between VJS and the endorsement of different conspiracy narratives might vary. Prior research and theorising indicate that VJS might be particularly relevant under conditions of high risks to the self (Baumert et al. 2020; Köhler et al. 2024) and when the situation is complex, risky and uncertain (Baumert et al. 2011; Gollwitzer and Rothmund 2009, 2011). Thus, it is plausible that VJS is more strongly associated with the endorsement of conspiracy narratives that are perceived to be more individually threatening, such as vaccine-related (versus climate change-related) conspiracy theories, as occurs in our data. Future research should address the moderating role of the idiosyncratic features of specific conspiracy beliefs in relation to VJS. Similarly, the conceptual and statistical differences between measures of specific conspiracy beliefs and measures intended to assess people’s general tendency to endorse conspiracy theories (Sutton et al. 2024; Sutton and Douglas 2020) could also explain this variation. Given that measures of general conspiracy beliefs arguably remove some of the idiosyncrasies of specific conspiracy theories (potentially those making individual victimhood more or less salient) and that they tend to show more normal distributions, the observation of stronger associations should be more likely.

### 5.1 Limitations and Future Research

Beyond its correlational nature, there are other limitations of this research, some of which refer to the measurement of VJS. One of these limitations is the conceptual overlap between the VJS scale and measures of relative deprivation (e.g., Callan et al. 2011; Obaidi et al. 2019), a construct known to correlate with conspiracy beliefs (e.g., Bilewicz et al. 2013; Gkinopoulos et al. 2023). This overlap is no coincidence, considering that theories of relative deprivation inspired the conceptualisation of justice sensitivity (Schmitt et al. 2005, 2010). This being said, relative deprivation refers to the subjective perception that ‘I’ (i.e., personal deprivation) or ‘we’ (group deprivation) get less than what I/we deserve (relative to others). Thus, social comparison lies at the heart of relative deprivation perceptions (e.g., Callan et al. 2011). VJS, however, reflects a latent fear of being exploited (Gollwitzer et al. 2013; Gollwitzer et al. 2015), that is, the anxious expectation that other people cannot be trusted. This distinction also reflects at the measurement level, where VJS and relative deprivation indeed share some conceptual ground—that is, the perception of, and often the dissatisfaction with, a disadvantaging social comparison—but still differ in a fundamental aspect: VJS explicitly emphasises the emotional reaction to the unfairness of being disadvantaged or exploited (e.g., ‘It makes me angry when others are undeservingly better off than me’; Schmitt et al. 2010), whereas measures of relative deprivation do not always stress this aspect (e.g., ‘I feel dissatisfied with what I have compared to what other people like me have’; Callan et al. 2011; see also Bilewicz et al. 2013). That VJS specifically involves the perception that one’s disadvantage is undeserved or unfair is important since one may feel deprived without necessarily feeling wronged. This distinction aligns with the core function of VJS in fostering a self-oriented sense of victimhood, which we have argued is especially relevant regarding the endorsement of conspiracy theories. Future research should nevertheless clarify to what extent the relative deprivation aspect (i.e., the perception of being disadvantaged) and/or the injustice aspect (i.e., the feeling of being wronged) of VJS contributes to the observed association.

Moreover, the use of short scales to assess both VJS and conspiracy beliefs in Study 2 can potentially entail different psychometric issues related to reliability and content validity. For example, although the Spearman–Brown’s estimate of the VJS scale was acceptable, the assessment of VJS with only two items could compromise the temporal and situational stability of the trait assessment (Baumert et al. 2014). Yet, results seem to replicate those of Study 1 with the original 10-item version of the *Justice Sensitivity Inventory* (Schmitt et al. 2010), which offers better psychometric properties and includes items that differ more from the concept of relative deprivation (e.g., ‘I cannot easily bear it when others profit unilaterally from me’). Regarding content validity, we intentionally limited the items used to assess specific conspiracy beliefs to science-related conspiracy theories due to their relationship with other science-related variables assessed through the main TISP survey. However, the consistent positive association between VJS and the measures of general conspiracy beliefs (i.e., conspiracy mentality and Lantian et al.’s [2016] scales) suggests that these results could likely generalise to other conspiracy beliefs.

Additionally, we acknowledge that there are limitations in using country-level indicators of direct violence (i.e., involvement in armed conflicts, state political terror) as moderators of the association between VJS and conspiracy beliefs. These indicators objectively capture contextual features that are often associated with processes of collective victimhood (Bilewicz 2022; Noor et al. 2017), but they do not capture the psychological experience of collective victimhood. Put differently, there might be countries where recent involvement in intergroup conflict or repression does not necessarily translate into a prevalent sense of collective victimhood (e.g., United States), and others where the sense of collective victimhood persists despite a lack of recent conflict or repression (e.g., Poland; Skrodzka and Vollhardt 2024). A more precise approach would involve the use of direct measures at the individual and/or country level of the subjective symbolic, transgenerational and culturally reinforced dimensions of collective victimhood (e.g., Skrodzka and Vollhardt 2024). However, we also note that applying such measures in crossnational research presents challenges, particularly due to the idiosyncrasies of a given context (e.g., Vollhardt et al. 2021), and therefore, potential violations of measurement invariance across contexts. In this regard, the use of objective countrylevel indicators, while imperfect, offers a degree of comparability and standardisation that is valuable for analyses across diverse cultural contexts.

## 6 Conclusion

Conspiracy beliefs are argued to be grounded in a sense of victimhood. While past research has focused on collective victimhood, the present investigation extends previous theorising by showing that the individual predisposition to perceive oneself as a victim of injustice is also associated with belief in conspiracy theories related to climate change and vaccines, as well as more general conspiracy narratives. We believe that the dispositional approach of the current research serves to extend the conceptual integration of group- and individual-level features of victimhood in the study of conspiracy beliefs. This could increase our understanding of a phenomenon that may not always arise in contexts of collective victimhood but that seems to align with individuals’ predisposition to perceive themselves as victims of injustice, including others’ evil and secret agendas.

## Figures and Tables

**Table 1:** Linear regression with conspiracy mentality as criterion, from secondary analyses of Altenmüller and Poppe (2025; Sample 2).

Predictor	*β* [95% CI]	*P*	sr^2^	*r*
VJS	0.15 [0.05, 0.26]	0.005	0.02	0.20**
Dispositional mistrust	0.18 [0.08, 0.28]	<0.001	0.03	0.23**
Intolerance of ambiguity	0.04 [-0.08, 0.15]	0.520	<0.01	0.11*
Need for control	0.06 [-0.04, 0.17]	0.255	<0.01	0.07
Political orientation	0.07 [-0.03, 0.17]	0.186	<0.01	0.12*
Observations		371/373		
*R^2^* [95% CI]		0.090 [0.03, 0.14]		

*Note: β* indicates the standardised regression weights. *p* represents the *p*-value for *β*. sr^2^ represents the semi-partial correlation squared. *r* represents the zero-order correlation. A significant *β*-weight indicates that the semi-partial correlation is also significant. Political orientation was measured from ‘left’ (lower values) to ‘right’ (higher values). Since two people did not state their political orientation, the regression results are based on *n* = 371 and the zero-order correlations are based on *n* = 373 for all other variables except political orientation.

* *p* < 0.05.

** *p* < 0.01.

**Table 2:** Sample size and descriptive statistics per country.

Country	ISO3	*N*	% Female	*M*_age_ [95% CI]	SD_age_	Range_age_	% HE
Australia	AUS	1437	48.50	45.53 [44.67, 46.40]	16.76	18−88	68.62
Austria	AUT	1036	51.16	44.94 [44.03, 45.86]	15.02	18−74	33.69
Bulgaria	BGR	499	49.90	44.57 [43.28, 45.85]	14.59	18−78	66.53
Chile	CHL	1009	50.55	44.49 [43.56, 45.42]	14.99	18−83	70.27
Colombia	COL	500	49.60	44.84 [43.53, 46.14]	14.85	18−89	68.80
Costa Rica	CRI	542	50.00	40.18 [39.06, 41.30]	13.25	18−76	59.04
Germany	DEU	2518	50.16	44.68 [44.10, 45.27]	15.00	18−90	33.64
Denmark	DNK	1072	50.28	45.53 [44.58, 46.47]	15.79	18−82	55.88
Greece	GRC	512	49.61	44.15 [42.89, 45.41]	14.48	18−76	68.80
Indonesia	IDN	1001	51.05	35.86 [35.20, 36.51]	10.52	18−71	79.82
Mexico	MEX	505	50.10	43.94 [42.68, 45.20]	14.37	18−81	62.97
New Zealand	NZL	1015	49.85	45.40 [44.40, 46.39]	16.14	18−91	70.54
Poland	POL	1274	48.90	44.55 [43.70, 45.40]	15.43	18−84	43.09
Russia	RUS	1523	50.56	44.21 [43.48, 44.93]	14.43	18−85	63.62
United States	USA	535	51.59	46.21 [44.86, 47.57]	15.98	18−79	65.61
Total sample		14,978	50.07	44.08 [43.84, 44.32]	15.15	18−91	57.00

*Note:* HE, higher education (e.g., university degree or higher education diploma).

**Table 3:** Preregistered multilevel models regressing different measures of conspiracy beliefs on victim justice sensitivity (VJS), unstandardised and centred within cluster (CWC).

Predictors	CB climate change				CB vaccines				CB general		
	*b* [95% CI]	*t*	*P* (Adj.p)		*b* [95% CI]	*t*	p (Adj. p)		*b* [95% CI]	*t*	*P*
Intercept	2.03 [1.83, 2.24]	21.03	<0.001		2.69 [2.47, 2.91]	26.32	<0.001		5.97 [5.61, 6.33]	35.75	<0.001
VJS (CWC)	0.10 [0.02, 0.19]	2.54	0.023 (0.023)		0.16 [0.07, 0.25]	3.87	0.002 (0.003)		0.33 [0.21, 0.45]	6.05	<0.001 (<0.001)
**Random effects**											
*σ* ^2^		0.93				1.10				3.36	
*τ*_00_		0 14 _country_iso_				0.15 _country_iso_				0 41 _country_iso_	
*τ*_11_		0 02 _country_iso.VJS_CWC_				0.02 _country_iso.VJS_CWC_				0.04 _country_iso.VJS_CWC_	
*ρ*_01_		0 81_country_iso_				0.38 _country_iso_				−0.10 _country_iso_	
ICC		0.16				0.15				0.13	
*N*		15 _country_iso_				15 _country_iso_				15 _country_iso_	
Observations		14,792				14,788				14,746	
Marginal *R*^2^/conditional *R*^2^		0.018/0.179				0.039/0.187				0.053/0.172	

*Note:* Adj. *p* represents *p*-values adjusted for multiple testing using Bonferroni–Holm corrections applied to the *n* = 3 tests of the association between VJS and the different measures of conspiracy beliefs.

**Table 4:** Multilevel models regressing different measures of conspiracy beliefs on victim justice sensitivity (VJS) centred within cluster (CWC)— within-country effect—and VJS country mean—between-country effect.

	CB climate change				CB vaccines				CB general		
Predictors	*b* [95% CI]	*t*	*P* (Adj. *p)*		*b* [95% CI]	*t*	*P* (Adj. *p*)		*b* [95% CI]	*t*	*P* (Adj. *p*)
Intercept	1.04 [−0.22, 2.30]	1.75	0.100		0.30 [−1.49, 2.09]	0.36	0.725		5.32 [1.66, 8.97]	3.14	0.008
VJS (CWC)	0.10 [0.02, 0.19]	2.52	0.024 (0.024)		0.16 [0.07, 0.25]	3.83	0.002 (0.004)		0.33 [0.21, 0.45]	6.03	<0.001 (<0.001)
VJS (country mean)	0.29 [−0.07, 0.66]	1.70	0.110 (0.220)		0.70 [0.18,1.22]	2.88	0.012 (0.037)		0.19 [−0.87, 1.25]	0.39	0.704 (0.704)
**Random effects**											
*σ* ^2^	0.93				1.10				3.36		
*τ*_00_	0.10 _country_iso_				0.10 _country_iso_				0.44 _country_iso_		
*τ*_11_	0.02 _country_iso.VJS_CWC_				0.02 _country_iso.VJS_CWC_				0.04 _country_iso.VJS_CWC_		
*ρ*_01_	0.75 _country_iso_				−0.06 _country_iso_				−0.18 _country_iso_		
ICC	0.14				0.12				0.13		
*N*	15 _country_iso_				15 _country_iso_				15 _country_iso_		
Observations	14,792				14,788				14,746		
Marginal *R^2^/* conditional *R*^2^	0.026/0.160				0.076/0.188				0.053/0.181		

*Note:* Adj. *p* represents *p*-values adjusted for multiple testing using Bonferroni–Holm corrections applied to the *n* = 3 tests of the association between VJS, VJS (country mean) and the different measures of conspiracy beliefs.

**Table 5:** Multilevel models including demographic variables as covariates.

Predictors	CB climate change				CB vaccines				CB general		
	*b* [95% CI]	*t*	*p*(Adj.*p*)		*b* [95% CI]	*t*	*p*(Adj.*p*)		*b* [95% CI]	*t*	*p*(Adj.*p*)
Intercept	1.07 [−0.28, 2.42]	1.68	0.111		0.50 [−1−29, 2.29]	0.60	0.558		6.02 [2.18, 9.85]	3.38	0.005
VJS (CWC)	0.08 [−0.01, 0.17]	1.87	0.082 (0.082)		0.14 [0.06, 0.23]	3.51	0.003 (0.007)		0.32 [0.19, 0.45]	5.23	<0.001 (<0.001)
VJS (country mean)	0.26 [−0.13, 0.65]	1.44	0.171 (0.341)		0.64 [0.12,1.16]	2.65	0.019 (0.057)		−0.01 [−1.12, 1.10]	−0.02	0.987 (0.987)
Age (CWC)	−0.00 [−0.01, −0.00]	−6.11	<0.001		−0.01 [−0.01, −0.01]	−8.87	<0.001		0.00 [0.00, 0.01]	2.10	0.036
Gender [Male]	0.17 [0.12, 0.21]	7.76	<0.001		−0.05 [−0.10, −0.00]	−2.02	0.043		0.02 [−0.07, 0.10]	0.35	0.725
Residence setting [Urban]	−0.03 [−0.08, 0.02]	−1.22	0.223		−0.04 [−0.09, 0.01]	−1.52	0.128		−0.14 [−0.23, −0.04]	−2.80	0.005
Political Orientation (CWC) Conservative vs. Liberal	0.15 [0.12, 0.17]	12.61	<0.001		0.12 [0.10, 0.15]	9.54	<0.001		0.15 [0.10, 0.20]	6.40	<0.001
Political Orientation (CWC) Right vs. Left	0.27 [0.24, 0.29]	21.33	<0.001		0.23 [0.21, 0.26]	16.81	<0.001		0.23 [0.18, 0.28]	9.05	<0.001
Religiosity (CWC)	0.08 [0.07, 0.10]	9.65	<0.001		0.09 [0.07, 0.11]	9.47	<0.001		0.11 [0.08, 0.15]	6.33	<0.001
**Random effects**											
*σ* ^2^		0.78				0.98				3.21	
*τ*_00_		0.15 _country_iso_				0.11 _country_iso_				0 47 _country_iso_	
*τ*_11_		0.02 _country_iso.VJS_CWC_				0.02 _country_iso.VJS_CWC_				0.05 _country_iso.VJS_CWC_	
*ρ*_01_		0.83 _country_iso_				0 99 _country_iso_				0.01 _country_iso_	
ICC		0.20				0.13				0.15	
*N*		15 _country_iso_				15 _country_iso_				15 _country_iso_	
Observations		11,221				11,219				11,207	
Marginal *R*^2^/conditional *R*^2^		0.185/0.352				0.186/0.294				0.098/0.233	

*Note:* Continuous variables were clustered within country (CWC). The number of observations for exploratory analyses including demographics is lower due to missing values. In particular, measures of political orientation showed a considerable percentage of participants (roughly 20%) who did not answer this question or responded ‘I don’t know,’ which we coded as a missing value. Adj. *p* represents *p*-values adjusted for multiple testing using Bonferroni–Holm corrections applied to the *n* = 3 tests of the association of VJS and VJS (country mean) with the different measures of conspiracy beliefs.

## Data Availability

The data that support the findings of this study are openly available in the Open Science Framework at https://osf.io/72dcn/.

## Footnotes

1 The correlation between Human Freedom Index and the Corruption Perception Index (Transparency International 2022), often related to conspiracy beliefs, was very high (*r* = 0.94), which indicated that these two indexes were capturing virtually the same country-level phenomenon (in fact, the Human Freedom Index includes some corruption items within its *Rule of Law—Criminal Justice* subfactor; Vásquez et al. 2022). Furthermore, the Corruption Perception Index shared higher correlations with GDP (*r* = 0.83) and the Gini index (*r* = −0.40), relative to the Human Freedom Index (*r* = 0.74 and *r* = −0.24, respectively), which led us to decide to examine Human Freedom Index only.
